# Prognostic Value of MicroRNA-15a in Human Cancers: A Meta-Analysis and Bioinformatics

**DOI:** 10.1155/2019/2063823

**Published:** 2019-04-02

**Authors:** Fei-ran Yang, Hui-jie Li, Ting-ting Li, Yu-feng Zhao, Zong-kai Liu, Xiu-rong Li

**Affiliations:** ^1^College of Traditional Chinese Medicine, Shandong University of Traditional Chinese Medicine, Jinan, Shandong, China; ^2^Department of Oncology, Affiliated Hospital of Shandong University of Traditional Chinese Medicine, Jinan, Shandong, China

## Abstract

**Background:**

Although several studies have proved the relationship between the prognostic value of miRNA-15a and different types of cancer, the result remains controversial. Thus, a meta-analysis was conducted to clarify the prognostic value of miRNA-15a expression level in human cancers.

**Methods:**

We enrolled appropriate literature by searching the databases of PubMed, Embase, and Web of Science. Subsequently, we extracted HRs and their 95% CIs and calculated pooled results of miRNA-15a for overall survival (OS) and disease-free survival (DFS). Besides, subgroup analysis, sensitivity analysis, and publication bias were also revealed in this study. We also further validated this meta-analysis using the Kaplan-Meier plotter database.

**Result:**

10 studies, including 1616 patients, were embraced in our meta-analysis. The result showed the lower expression of miRNA-15a significantly predicted adverse OS (HR=2.17, 95% CI: 1.41-3.34), but there is no significant association between the expressing level and DFS in cancer patient (HR=2.04, 95% CI: 0.60-6.88). Based on Kaplan-Meier plotter database, we found the same results in bladder Carcinoma, head-neck squamous cell carcinoma, liver hepatocellular carcinoma, lung squamous cell carcinoma, pancreatic ductal adenocarcinoma, rectum adenocarcinoma, stomach adenocarcinoma, and uterine corpus endometrial carcinoma, but opposite results were found in cervical squamous cell carcinoma and esophageal carcinoma.

**Conclusion:**

Low expressing levels of miRNA-15a indicated poor OS, while miRNA-15a can be used as a prediction biomarker in different cancer types.

## 1. Introduction

MicroRNAs (miRNAs), endogenous noncoding single‐stranded RNAs with 20-25 nucleotides in length, regulate gene expression by binding with imperfect complementarity to the 3late gene expression at specific mRNAs. They may regulate approximately 60% genes in human protein-coding [1, 2]. Previous studies have demonstrated that miRNAs play important roles in various fundamental and biological processes including cell differentiation, proliferation, metabolism, differentiation, and apoptosis [3, 4]. It is well documented that some miRNAs act as tumor suppressors or oncogenes, which is frequently down-/upregulated in malignancies. [5] Because of their non-invasive as well as unique expression patterns, miRNAs were accepted as good diagnostic or prognostic biomarker in human cancer research.

MiRNA-15a is an important part of the miRNA-15 family [6], which has been reported to function as a tumor suppressor in carcinogenesis via targeting oncogenes. For example, anti-apoptotic BCL2 in chronic lymphocytic leukemia (CLL) and other cancers [7] and BMI1 in gastric cancer as well as pancreatic cancer act as a stem cell marker and promoter of migration and invasion [8]. MiRNA-15a also targets MCL1, WNT3A, and MCL1 [9]. Downregulation of miRNA-15a has recently been reported in patients with CLL [7], non-small-cell lung cancer [10], prostate cancer [11], ovarian cancer [12], and hepatocellular carcinoma [13]. As an important member of miRNA-15/16 cluster, the influence of miRNA-15a on the prognosis of colorectal cancer [14–17], osteosarcoma [18], esophageal squamous cell carcinoma [19], glioma [20], breast cancer [21], cholangiocarcinoma [22], and multiple myeloma [23] has been reported. However, the results were insignificant or opposite. Thus, we conducted this meta-analysis to comprehensively assess the prognostic value of miRNA-15a expression level in human cancers.

## 2. Materials and Methods

### 2.1. Search Strategy

**Figure 1:**
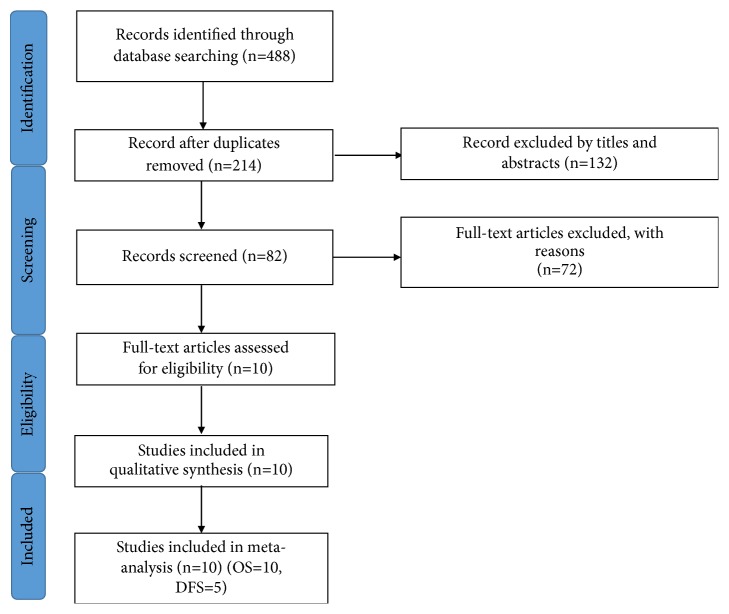
Flow diagram of the study selection process. OS, overall survival; DFS, disease-free survival.

The meta-analysis according to the guidelines of the Preferred Reporting Items for Systematic Reviews and Meta-Analysis (PRISMA) statement and methods. [24] We retrieved some online databases consisting of PubMed, Embase, and Web of Science to find eligible studies till October 2018. The articles were searched by using the following keywords: microRNA-15a, miRNA-15a, and miR-15a combined with prognosis, prognostic or survival. We use the following aspects to identify if the literature is duplicated, including author names, institutions, clinical trial registration number, numbers of participants, baseline data, and specific details of the interventions. For the literature that has been reported many times by the same author, we select the latest and most complete inclusion. Additionally, we manually searched previous reviews and the references list to the literature included in our study to find out additional relevant studies.  Figure 1 showed the flow diagram of the literature selection process.

### 2.2. Data Extraction

Each study was revised by two reviewers (FRY and TTL) followed a standard data extraction form. Discrepancies were solved by fully discussing with HJL or further reviewed by XRL. Eligible studies must comply with the following criteria: (1) study about the correlation of miRNA-15a with any type of cancer prognosis; (2) publication details: disease name, publication year, and first author's last name; (3) the effect size being evaluated using multivariate HRs with 95% confidence intervals (CI) for OS or DFS, if the HRs cannot be obtained directly from the original studies, data were calculated by Kaplan-Meier curves according to the method provided by Tierney et al. [25] Studies were excluded if one of the following criteria was met: (1) review, case report, abstracts, or letters to editor; (2) duplicate articles; (3) animal models or cell lines as research subject; (4) survival data lacking or being unable to calculate them.

### 2.3. Quality Assessment

All the enrolled literature was evaluated by Newcastle-Ottawa scale. This quality evaluation scale of literature contains the following three aspects: study groups selection, comparison of study groups, and measuring of outcomes. The score of this scale is ranged from 0 to 9.

### 2.4. Bioinformatics Analysis

To further validate and complement this meta-analysis, we used the Kaplan-Meier plotter database and miRpower for pan-cancer tool to analyze the relationship between miRNA-15a expression and OS of human cancers (http://kmplot.com/analysis/index.php?p=service&cancer=pancancer_mirna). [26] If the* P*-value is less than 0.05, it is considered statistically significant.

### 2.5. Statistical Analysis

The pooled HRs were calculated by HRs with 95% CIs. Heterogeneity between pooled studies was evaluated by Cochran's Q test and Higgin's* I*^2^ statistic. We preferred to use fixed-model if there is no significant heterogeneity (P>0.1 and* I*^*2*^*<50%)*; otherwise, we used the random-effect model. Publication bias was evaluated using the funnel plot with Begg's statistical test. We use the STATA software for statistical analysis (STATA Corporation, College Station, USA, version 14.0).

## 3. Results

### 3.1. Features of Studying

We used the search strategy provided above to retrieve 488 records from online databases. By manually reviewing titles and abstracts, we rejected duplicates and unavailable literature. Then 82 articles were selected for full-text browsing. Finally, ten studies were included and there was no additional article by consulting the cross-references. Ten studies consisted of 1616 samples from China, US, Australia, Greece, Japan, and Thailand and were included to evaluate the relationship between miRNA-15a expression and cancer prognosis. Eight of the 10 studies detected the expression of miRNA-15a in tumor tissue and another two studies [19, 23] detected in serum and bone marrow, respectively. To measure the expression of miRNA-15a, all of the studies used qRT-PCR. Six of 10 studies used the exact value of miRNA expression as the cut-off value, two studies used the median value [17, 20], and the remaining two studies did not provide relevant information. [21, 22] For survival assessment criteria, all incorporated literature used OS and five studies used DFS. [16, 17, 19–21] The HRs and 95% CIs were directly acquired in seven studies, evaluation from the survival curve in three records. [14, 15, 19] The main features of the available studies have been listed in Table 1. Summary of HRs and their 95% CI are shown in Table 2.

### 3.2. Qualitative Assessment

After using Newcastle–Ottawa scale to assess the quality of studies, the study quality score >6 was included. The main characteristics of the eligible studies are summarized in Table 3.

### 3.3. Meta-Analysis Results

**Figure 2:**
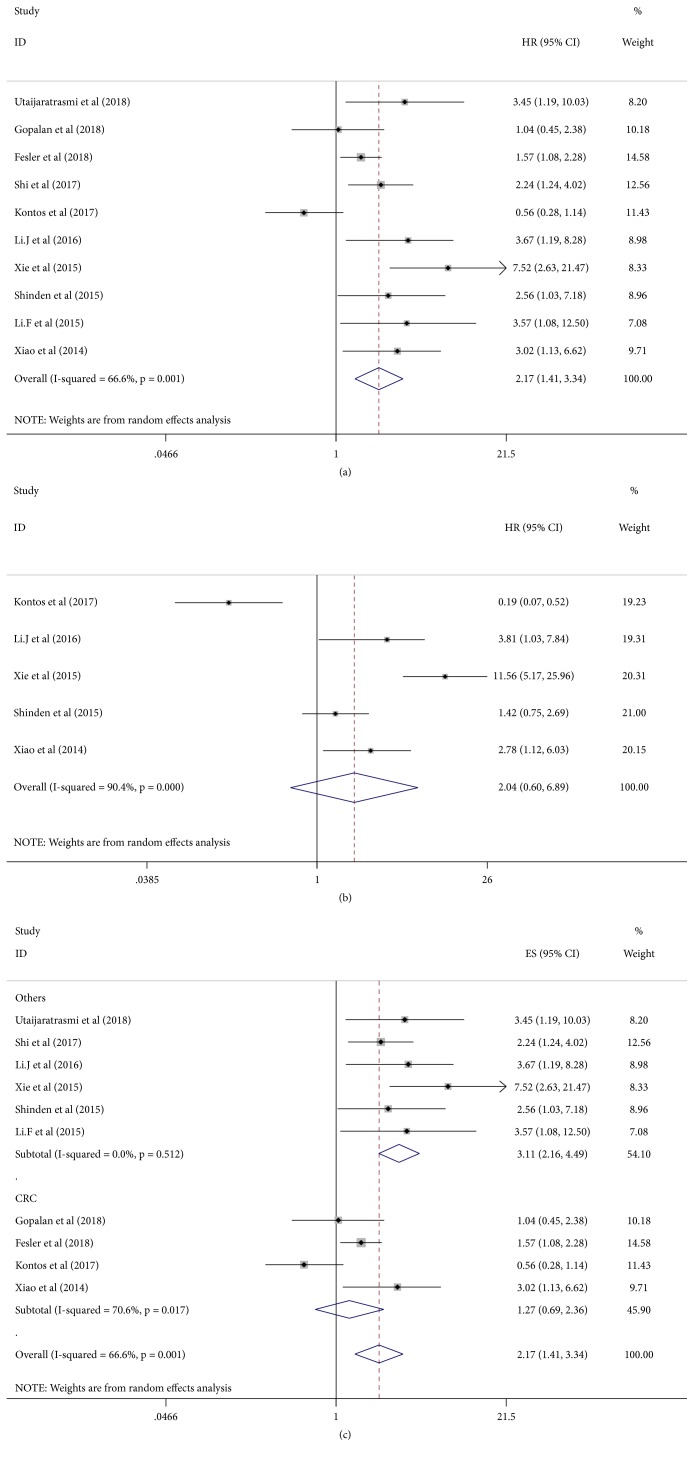
Forest plot of overall survival analysis and disease-free survival analysis.* Note*. (a) Meta-analysis of miRNA-15a expression and overall survival. (b) Meta-analysis of miRNA-15a expression and disease-free survival. (c) Meta-analysis of miRNA-15a expression and overall survival in different kinds of cancers.

All the studies with 1616 cancer patients we have included were involved in OS analysis. Because of high heterogeneity (*I*^2^ =66.6%, P=0.001), we chose a random-effect model to compare between low expression of miRNA-15a and high expression. The result showed that lower expression of miRNA-15a indicating adverse OS (HR=2.17, 95% CI: 1.41-3.34; Figure 2(a)), and the results are statistically significant (P<0.001). Five studies with 772 cancer patients were involved in DFS analysis; a pooled HRs and its 95% CIs were conducted with random-effect model due to the high heterogeneity (*I*^2^ =90.4%, P=0.001). We found that there is no significant association between the expressing level and DFS in cancer patients (HR=2.04, 95% CI: 0.60-6.88; Figure 2(b).

In this study, we performed a subgroup analysis based on the main characteristics of the included studies. The results indicated that the prognosis value of miRNA-15a was significant in tissue specimen (HR=1.97, 95% CI: 1.22-3.19), serum or bone marrow specimen (HR=3.63, 95% CI: 1.70-7.76), and Asia patients (HR=3.10, 95% CI: 2.21-4.34). The association between downregulation of micro-RNA15a expression and shorter OS time was not obvious in the subgroup of European and colorectal cancer patients. (Table 4)

### 3.4. Survival Analysis of Human Cancers through the Kaplan–Meier Plotter Database

**Figure 3:**
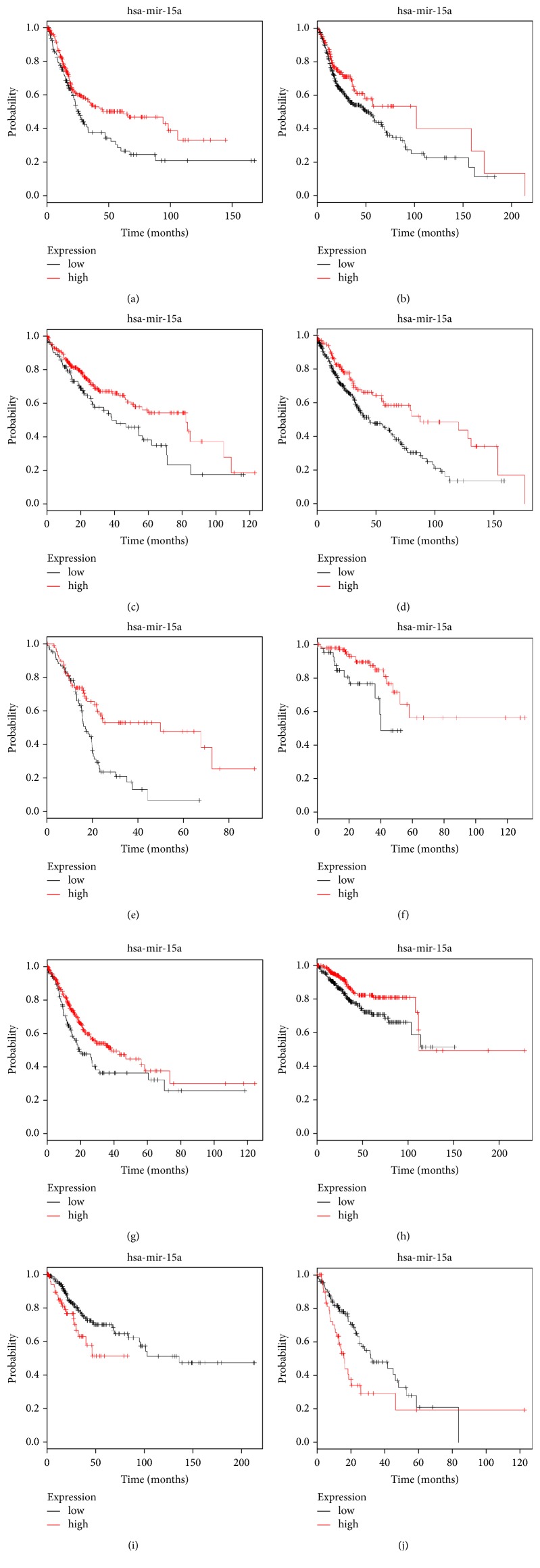
Kaplan–Meier survival curves for cancer patients, stratified by miRNA-15a expression levels. Note: (a) BLCA, (b) HNSC, (c) LIHC, (d) LUSC, (e) PAAD, (f) READ, (g) STAD, (h) UCEC, (i) CSCC, and (j) ESCA.

Survival analysis was performed through the Kaplan–Meier plotter database to validation for the results of meta-analysis which included 7385 patients with various types of cancer (Table 5). The results showed that although the probability of death in the low-expression group was 1.165 times higher than that in the high-expression group, there was no statistical difference (HR=1.165, 95%CI 0.95-1.44;* P*=0.150). However, in the survival analysis of a single cancer types, we found that low-expression miRNA-15a was significantly associated with worse OS in bladder Carcinoma (HR=1.49, 95%CI 1.11-2.00;* P*=0.0081), head-neck squamous cell carcinoma (HR=1.43, 95%CI 1.04-1.96;* P*=0.027), liver hepatocellular carcinoma (HR=1.54, 95%CI 1.08-2.22;* P*=0.017), lung squamous cell carcinoma (HR=1.69, 95%CI 1.22-2.38;* P*=0.0014), pancreatic ductal adenocarcinoma (HR=2.22, 95%CI 1.43-3.45;* P*=0.0002), rectum adenocarcinoma (HR=2.63, 95%CI 1.19-5.88;* P*=0.013), stomach adenocarcinoma (HR=1.52, 95%CI 1.10-2.08;* P*=0.011), and uterine corpus endometrial carcinoma (HR=1.69, 95%CI 1.10-2.63;* P*=0.015), whereas the results were opposite in cervical squamous cell carcinoma (HR=0.55, 95%CI 0.32-0.93;* P*=0.026) and esophageal carcinoma (HR=0.53, 95%CI 0.34-0.85;* P*=0.0072) (Figure 3). In the other caner types, there is no significant association between the expression of miRNA-15a and human cancers. Thus, most of the results from Kaplan–Meier plotter database were consistent with our meta-analysis.

### 3.5. Sensitivity Analysis

**Figure 4:**
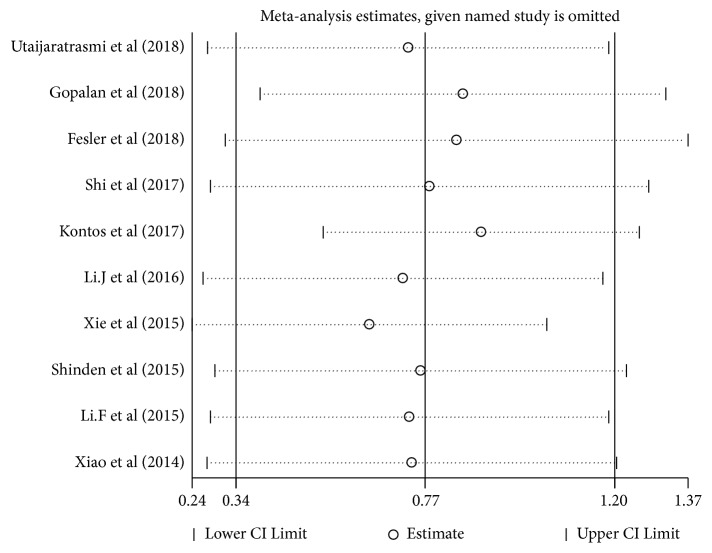
Result of sensitivity analyses by omitting one study in each turn.

To assess the stability of the meta-analysis results, we carried out sensitivity analysis by excluding studies one by one. After each study is excluded, the pooled HR was recalculated. The result showed that the stability of the entire study was not affected by a certain study (Figure 4).

### 3.6. Publication Bias Assessment

**Figure 5:**
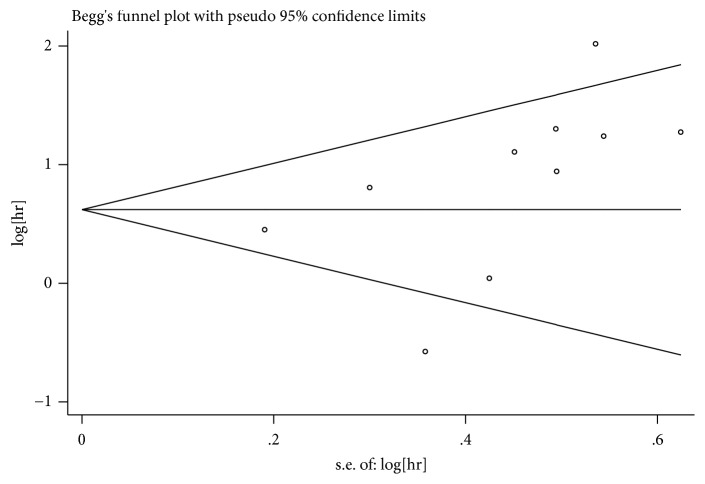
Funnel plot of miRNA-15a and overall survival.

Publication bias for the OS meta-analysis among included studies was evaluated by Bgger's funnel plot (Figure 5). The funnel plot was basically symmetrical and the P-value of Bgger's regression intercepts was 0.107. Consequently, there was no significant risk of publication bias in this meta-analysis.

## 4. Discussion

As a class of tiny regulatory RNA molecules, miRNA plays an important role in gene expression and diverse biological processes. They can blind to the 3′UTR and 5′UTR of their target mRNAs. A large number of studies have demonstrated that tumor invasion and metastasis are regulated by miRNAs, and they are used to analyze the prognosis of a variety of tumors. For example, Wang et al. have demonstrated that overexpression of miRNA-34a significantly predicted good OS in cancer patients. [27] Zhang et al. found that downregulated miRNA-183 was notably indicated for poor OS. [28] Hence, miRNAs have the potential to be used as a biomarker for analyzing tumor prognosis.

MiRNA-15a was the first miRNA to be discovered as a tumor suppressor in CLL. [29] MiRNA-15a belongs to the miRNA-15 family, which is located on chromosome 13 (13q14) and consists of miRNA-15a/b, miRNA-16-1, miRNA-16-2, miRNA-497, and miRNA-195. [6] Furthermore, they are located in the intron of DLEU2, which is a long non-coding RNA (lncRNA) gene. [30] Plenty of researches have revealed that the expression level of miRNA-15a is adjusted by many factors, such as transcription factors and epigenetic and chromosomal deletions. Cutrona et al. discovered that the chromosome 13q14 deletion is related to a significant downregulation of miRNA-15a and the pathogenesis of CLL. [31] The mechanism by which miRNA-15a promotes tumor progression remains complex; therefore, more researches are needed to disclose it. Some researches have discovered that miRNA-15a inhibits cell proliferation by modulating many specific targets. For example, cyclin has been revealed to play critical roles in cell proliferation. In CLL, breast cancer, and lung cancer, DLEU2 downregulates cyclins by upregulating miRNA-15a, leading to cell cycle arrest in the G1 to G0 phase. [32, 33] Related studies have shown that the pathogenesis of many cancers is closely related to the abnormal regulation of apoptosis. Bcl-2 has been shown as a critical gene in cell apoptosis and it is a significant target of miRNA-15a in CLL. Deletion of 13q14 resulted in downregulation of miRNA-15a and overexpression of Bcl-2. [34] As we know, EMT (epithelial–mesenchymal transition) is an important biological process for malignant tumor cells to acquire the ability of migration and invasion, and Twist1 as a transcription factor plays a decisive role in the regulation of the EMT process. Interestingly, upregulated miRNA-15a inhibits the activity of EMT-related genes, such as N-cad, E-cad, and Twist1. In gastric cancer and NSCLC tissues, miRNA-15a was significantly downregulated, while Twist1 gene and its regulated proteins were significantly increased. [35]

Recently, a large number of reports indicate that there is a correlation between the expression of miRNA-15a and the prognosis of various kinds of cancers. To identify the prognostic value of miRNA-15a, we performed this meta-analysis of 10 studies and 1616 patients with 7 cancers. We found that lower expression of miRNA-15a was associated with shorter OS. In other words, patients with higher expression of miRNA-15a have longer OS time than low expression levels. The expression levels of miRNA-15a are downregulated in most cancer types, and they may function as tumor suppressor genes. But obvious heterogeneity was found in our meta-analysis, so we conducted a subgroup analysis by cancer types, race, publication year, and material of miRNA-15a. In the subgroup of cancer types, we found significant heterogeneity in CRC group (*I*^*2*^=70.6,* P*=0.017). When we removed the study of Kontos et al. from the subgroup of colorectal cancer types, no significant heterogeneity was found. In Kontos et al.'s study, low expression group significantly reduced risk of death, which was contrary to the other three studies, but it was not statistically significant (HR=0.56, 95%CI 0.28-1.14;* P*=0.11). Although there are significant statistical differences in the analysis of DFS (HR=0.19, 95%CI 0.07-0.52; P=0.001), OS is still the best indicator for prognosis in clinical practice. Therefore, we concluded that the main source of heterogeneity is the study by Kontos et al. In addition, we performed survival analysis of human cancers through the Kaplan–Meier plotter database and found that the results of esophageal carcinoma and breast cancer were contrary to our meta-analysis; we analyze the reasons that lead to this result which may have the following aspects: (1) the sample size is too small, for esophageal carcinoma, only 184 cases in the database and 106 cases in the meta-analysis. For breast cancer, comparing 1076 cases in the database, there is only one literature and 230 cases in the meta-analysis. (2) Pathological classification of esophageal cancer is not given in the database, which may also be the cause of inconsistent results. Interestingly, we found in the database that lower expression of miRNA-15a significantly predicted adverse OS in CRC, which is inconsistent with the result of our meta-analysis. However, considering the survival analysis of CRC is still controversial in our meta-analysis, so there should be more clinical studies in the future to discover the relationship between miRNA-15a and the prognosis of CRC. All in all, through our meta-analysis, we found that miRNA-15a may be an important biomarker for predicting the clinical outcome of cancer patients.

To the best of our knowledge, there was no previous meta-analysis of the relationship between miRNA-15a expression levels and the prognosis of cancer patients. Nevertheless, our research has some limitations. Firstly, only 10 studies with 1616 patients were included in our analysis; this may make the result inaccurate, and the reason for the heterogeneity may be because the sample size is too small. Secondly, the design of studies, cut-off value, and measure methods were distinct in different researches, and these factors may have a certain impact on the results of our analysis. Third, we cannot find the HRs and their 95% CIs directly from some literature; thus, we had to estimate results from the Kaplan-Meier curve. This reduced the credibility of our results. Fourth, histopathology is the gold standard for tumor diagnosis, because of its high accuracy. In clinical applications, blood samples were easier to obtain than tissues. Therefore, in the future researches, increasing blood sample applications can provide evidence for clinical diagnosis and treatment in the absence of tissue samples case.

## 5. Conclusion

As shown in our meta-analysis and bioinformatics, low expression of miRNA-15a may indicate poor OS in cancer patients. But there is no significant association between the expression level and DFS in patient with cancer and there is also controversy in esophageal cancer, breast cancer, and colorectal cancer. Further clinical studies are needed to demonstrate the association between miRNA-15a and cancer prognosis as well as treatment efficiency.

## Figures and Tables

**Table 1:** The main characteristics of included 10 studies.

Study	Year	Region	Study design	Disease	N	Stage	Sample	miRNA-15a	Cut-off	Survival analysis	Follow-up Time(month)
								assay			
Utaijaratrasmi	2018	Thailand	R	ICC	72	I-III	Tissue	qRT-PCR	NR	RE	Median 36
Gopalan et al	2018	Australia	R	CRC	124	II-IV	Tissue	qRT-PCR	2^−Δct^	SC	Median 78
Fesler et al	2018	US	R	CRC	431	II-IV	Tissue	qRT-PCR	0.65	SC	Up to 120
Shi et al	2017	China	R	OSM	127	I-III	Tissue	qRT-PCR	2^−ΔΔct^	SC	Median 47.5
Kontos et al	2017	Greece	R	CRC	182	I-IV	Tissue	qRT-PCR	0.54	RE	Median 33
Li.J et al	2016	China	R	ESCC	106	I-III	Serum	qRT-PCR	2^−ΔΔct^	RE	Median 28
Xie et al	2015	China	R	Glioma	128	I-IV	Tissue	qRT-PCR	Median	RE	Median 42.8
Shinden et al	2015	Japan	R	BC	230	I-IV	Tissue	qRT-PCR	NR	RE	Up to 60
Li.F et al	2015	China	R	MM	90	NR	Bone marrow	qRT-PCR	Median	RE	Median 15
Xiao et al	2014	China	R	CRC	126	I-IV	Tissue	qRT-PCR	Median	RE	Median 74

R, retrospective; BC, breast cancer; ICC, cholangiocarcinoma; CRC, colorectal cancer; OSM, osteosarcoma; ESCC, esophageal squamous cell carcinoma; MM, multiple myeloma; qRT-PCR, quantitative reverse transcription-polymerase chain reaction; RE, reported; SC: survival curve; NR, not reported.

**Table 2:** Summary of HRs and their 95% CI.

Study	Year	Region	Disease	HR	OS	HR	DFS
					95% CI		95% CI
Utaijaratrasmi	2018	Thailand	ICC	3.452	1.188-10.030	NR	NR
Gopalan et al	2018	Australia	CRC	1.04	0.45-2.38	NR	NR
Fesler et al	2018	US	CRC	1.57	1.08-2.28	NR	NR
Shi et al	2017	China	OS	3.281	2.901-3.525	NR	NR
Kontos et al	2017	Greece	CRC	0.56	0.28-1.14	0.185	0.066-0.518
Li.J et al	2016	China	ESCC	3.668	1.193-8.283	3.808	1.032-7.838
Xie et al	2015	China	Glioma	7.52	2.63-21.47	11.56	5.17-25.96
Shinden et al	2015	Japan	BC	2.56	1.03-7.18	1.42	0.75-2.69
Li.F et al	2015	China	MM	3.57	1.08-12.50	NR	NR
Xiao et al	2014	China	CRC	3.016	1.129-6.616	2.782	1.122-6.031

DFS, disease-free survival; HR, hazard ratio; OS, overall survival.

**Table 3:** Quality assessment based on the newcastle–Ottawa scale.

Study	Year	Selection	Comparability	Outcome	Total score
Utaijaratrasmi	2018	3	2	3	8
Gopalan et al	2018	4	2	2	8
Fesler et al	2018	3	1	2	6
Shi et al	2017	3	2	2	7
Kontos et al	2017	3	2	2	7
Li.J et al	2016	3	2	2	7
Xie et al	2015	4	2	2	8
Shinden et al	2015	3	2	2	7
Li.F et al	2015	3	2	3	8
Xiao et al	2014	3	2	2	7

**Table 4:** Meta-analysis of overall survival and subgroup analysis.

Subgroup	No of studies	HR (95% CI)	Model	Heterogeneity	
				*I* ^2^ (%)	P-value
Race					
Asian	6	3.10 (2.21-4.34)	random	0	0.640
European l	4	1.02 (0.53-1.96)	random	69.8	0.037
Year					
>2016	5	1.43 (0.85-2.41)	random	67.9	0.014
≦2016	5	3.67 (2.34-5.74)	random	0	0.642
Material					
Tissue	8	1.97 (1.22-3.19)	random	70.4	0.001
Serum/bone marrow	2	3.63 (1.70-7.76)	random	0	0.973
Cancer type					
Colorectal cancer	4	1.27 (0.69-2.36)	random	70.6	0.017
Other type of cancer	6	3.11 (2.16-4.49)	random	0	0.512

**Table 5:** HRs and 95%CIs of miRNA-15a downregulation in human cancers based on Kaplan-Meier plotter database.

Cancer types	Sample size	HR (95% CI)	*P*-value
Human cancers	7385	1.17 (0.95-1.44)	0.150
BLCA	408	1.49 (1.11-2.00)	0.0081
BRCA	1076	0.83 (0.58-1.16)	0.27
CSCC	307	0.55 (0.32-0.93)	0.026
ESCA	184	0.53 (0.34-0.85)	0.0072
HNSC	522	1.43 (1.04-1.96)	0.027
KIRC	516	0.78 (0.55-1.09)	0.14
KIRP	290	1.59 (0.84-3.03)	0.15
LIHC	371	1.54 (1.08-2.22)	0.017
LUAD	504	1.23 (0.92-1.67)	0.16
LUSC	472	1.69 (1.22-2.38)	0.0014
OC	485	0.82 (0.65-1.04)	0.10
PAAD	178	2.22 (1.43-3.45)	0.0002
PCPG	179	0.14 (0.02-1.20)	0.038
READ	160	2.63 (1.19-5.88)	0.013
SARC	259	0.69 (0.46-1.04)	0.075
STAD	431	1.52 (1.10-2.08)	0.011
THCA	506	4.17 (0.54-33.33)	0.140
UCEC	537	1.69 (1.10-2.63)	0.015

BLCA, bladder Carcinoma; BRCA, breast cancer; CSCC, cervical squamous cell carcinoma; ESCA, esophageal carcinoma; HNSC, head-neck squamous cell carcinoma; KIRC, kidney renal clear cell carcinoma; KIRP, kidney renal papillary cell carcinoma; LIHC, liver hepatocellular carcinoma; LUAD, lung adenocarcinoma; LUSC, lung squamous cell carcinoma; OC, ovarian cancer; PAAD, pancreatic ductal adenocarcinoma; PCPG, pheochromocytoma and paraganglioma; READ, rectum adenocarcinoma; SARC, sarcoma; STAD, stomach adenocarcinoma; THCA, thyroid carcinoma; UCEC, uterine corpus endometrial carcinoma.

## References

[1] Heneghan H. M., Miller N., Kerin M. J. (2010). MiRNAs as biomarkers and therapeutic targets in cancer. *Current Opinion in Pharmacology*.

[2] Esteller M. (2011). Non-coding RNAs in human disease. *Nature Reviews Genetics*.

[3] Bartel D. P. (2004). MicroRNAs: genomics, biogenesis, mechanism, and function. *Cell*.

[4] Kloosterman W. P., Plasterk R. H. A. (2006). The diverse functions of microRNAs in animal development and disease. *Developmental Cell*.

[5] Esquela-Kerscher A., Slack F. J. (2006). Oncomirs—microRNAs with a role in cancer. *Nature Reviews Cancer*.

[6] Hullinger T. G., Montgomery R. L., Seto A. G. (2012). Inhibition of miR-15 protects against cardiac ischemic injury. *Circulation Research*.

[7] Cimmino A., Calin G. A., Fabbri M. (2005). miR-15 and miR-16 induce apoptosis by targeting BCL2. *Proceedings of the National Acadamy of Sciences of the United States of America*.

[8] Guo S., Xu X., Tang Y. (2014). MiR-15a inhibits cell proliferation and epithelial to mesenchymal transition in pancreatic ductal adenocarcinoma by down-regulating Bmi-1 expression. *Cancer Letters*.

[9] Aqeilan R. I., Calin G. A., Croce C. M. (2010). *miR-15a* and *miR-16-1* in cancer: discovery, function and future perspectives. *Cell Death & Differentiation*.

[10] Bandi N., Vassella E. (2011). *MiR-34a* and *miR-15a/16* are co-regulated in non-small cell lung cancer and control cell cycle progression in a synergistic and Rb-dependent manner. *Molecular Cancer*.

[11] Musumeci M., Coppola V., Addario A. (2011). Control of tumor and microenvironment cross-talk by miR-15a and miR-16 in prostate cancer. *Oncogene*.

[12] Bhattacharya R., Nicoloso M., Arvizo R. (2009). MiR-15a and MiR-16 control Bmi-1 expression in ovarian cancer. *Cancer Research*.

[13] Huang Y.-H., Lin K.-H., Chen H.-C. (2012). Identification of postoperative prognostic microRNA predictors in hepatocellular carcinoma. *PLoS ONE*.

[14] Xiao G., Tang H., Wei W., Li J., Ji L., Ge J. (2014). Aberrant expression of microRNA-15a and microRNA-16 synergistically associates with tumor progression and prognosis in patients with colorectal cancer. *Gastroenterology Research and Practice*.

[15] Kontos C. K., Tsiakanikas P., Avgeris M., Papadopoulos I. N., Scorilas A. (2017). miR-15a-5p, a novel prognostic biomarker, predicting recurrent colorectal adenocarcinoma. *Molecular Diagnosis & Therapy*.

[16] Fesler A., Liu H., Ju J. (2018). Modified miR-15a has therapeutic potential for improving treatment of advanced stage colorectal cancer through inhibition of BCL2, BMI1, YAP1 and DCLK1. *Oncotarget *.

[17] Gopalan V., Ebrahimi F., Islam F. (2018). Tumour suppressor properties of miR-15a and its regulatory effects on BCL2 and SOX2 proteins in colorectal carcinomas. *Experimental Cell Research*.

[18] Shi J., Fu Q., Yang P., Liu H., Ji L., Wang K. (2018). Downregulation of microRNA-15a-3p is correlated with clinical outcome and negatively regulates cancer proliferation and migration in human osteosarcoma. *Journal of Cellular Biochemistry*.

[19] Li J., Li M., Gao F., Ge X. (2017). Serum microRNA-15a level acts as a potential diagnostic and prognostic biomarker for human esophageal squamous cell carcinoma. *Cancer Biomarkers*.

[20] Xie T., Liu P., Chen L. (2015). MicroRNA-15a down-regulation is associated with adverse prognosis in human glioma. *Clinical and Translational Oncology*.

[21] Shinden Y., Akiyoshi S., Ueo H. (2015). Diminished expression of MiR-15a is an independent prognostic marker for breast cancer cases. *Anticancer Reseach*.

[22] Utaijaratrasmi P., Vaeteewoottacharn K., Tsunematsu T. (2018). The microRNA-15a-PAI-2 axis in cholangiocarcinoma-associated fibroblasts promotes migration of cancer cells. *Molecular Cancer*.

[23] Li F., Xu Y., Deng S. (2015). MicroRNA-15a/16-1 cluster located at chromosome 13q14 is down-regulated but displays different expression pattern and prognostic significance in multiple myeloma. *Oncotarget *.

[24] Moher D., Liberati A., Tetzlaff J., Altman D. G. (2010). Preferred reporting items for systematic reviews and meta-analyses: the PRISMA statement. *International Journal of Surgery*.

[25] Tierney J. F., Stewart L. A., Ghersi D., Burdett S., Sydes M. R. (2007). Practical methods for incorporating summary time-to-event data into meta-analysis. *Trials*.

[26] Lánczky A., Nagy Á., Bottai G. (2016). miRpower: a web-tool to validate survival-associated miRNAs utilizing expression data from 2178 breast cancer patients. *Breast Cancer Research and Treatment*.

[27] Wang J., Dan G., Zhao J. (2015). The predictive effect of overexpressed miR-34a on good survival of cancer patients: A systematic review and meta-analysis. *OncoTargets and Therapy*.

[28] Zhang X., Pan S., Yan J., Xu G. (2018). The prognostic value of microRNA-183 in human cancers. *Medicine*.

[29] Cho W. C. S. (2007). OncomiRs: the discovery and progress of microRNAs in cancers. *Molecular Cancer*.

[30] Lindner S. E., Lohmüller M., Kotkamp B. (2017). The miR-15 family reinforces the transition from proliferation to differentiation in pre-B cells. *EMBO Reports*.

[31] Cutrona G., Matis S., Colombo M. (2017). Effects of miRNA-15 and miRNA-16 expression replacement in chronic lymphocytic leukemia: Implication for therapy. *Leukemia*.

[32] Chen C.-Q., Chen C.-S., Chen J.-J. (2013). Histone deacetylases inhibitor trichostatin A increases the expression of Dleu2/miR-15a/16-1 via HDAC3 in non-small cell lung cancer. *Molecular and Cellular Biochemistry*.

[33] Luo Q., Li X., Li J. (2013). MiR-15a is underexpressed and inhibits the cell cycle by targeting *CCNE*1 in breast cancer. *International Journal of Oncology*.

[34] Pekarsky Y., Croce C. M. (2014). Role of miR-15/16 in CLL. *Cell Death Differentiation*.

[35] Wang T., Hou J., Li Z. (2017). miR-15a-3p and miR-16-1-3p negatively regulate twist1 to repress gastric cancer cell invasion and metastasis. *International Journal of Biological Sciences*.

